# Stable isotopes reveal intensive pig husbandry practices in the middle Yellow River region by the Yangshao period (7000–5000 BP)

**DOI:** 10.1371/journal.pone.0257524

**Published:** 2021-10-05

**Authors:** Quan Zhang, Yanfeng Hou, Xinwei Li, Amy Styring, Julia Lee-Thorp

**Affiliations:** 1 School of Archaeology, University of Oxford, Oxford, United Kingdom; 2 Henan Provincial Institute of Cultural Heritage and Archaeology, Zhengzhou, China; 3 Institute of Archaeology, Chinese Academy of Social Sciences, Beijing, China; University at Buffalo - The State University of New York, UNITED STATES

## Abstract

It is well-known that pigs (*Sus scrofa*) were domesticated very early in Neolithic China, but far less is known about the processes by which pig husbandry intensified so that pork became the most important animal protein for humans are less clear. Here, we explore pig feeding practices using the carbon and nitrogen isotope composition of bone collagen, focusing on developments in pig husbandry during the Yangshao period (7000–5000 BP) in the middle Yellow River region of China, and at the site of Xipo (5800–5000 BP) in particular. The results show that the diets of domestic pigs at Xipo were dominated by millet foods. Comparisons with other Yangshao sites in the region show a trend of increasing millet foddering for pigs throughout the Yangshao period. These results, and comparisons of the isotopic data for pigs against those for humans from the Xipo cemetery (5300–5000 BP), suggest that pigs were closely managed by humans. The evidence points to an intensification of Neolithic pig husbandry in the middle Yellow River region from this period.

## 1. Introduction

It is widely acknowledged that a combination of domesticated crops and animals provided the subsistence basis and fostered human population growth and the rise of social complexity in Neolithic farming societies [1–3]. In Neolithic China, domestication and husbandry of pigs (*Sus scrofa*) came to provide the primary domestic animal protein source for humans [4–6]. However, the processes of development and intensification of pig husbandry remain underexplored. This is partly because the ecological and behavioural plasticity of pigs means they are adaptable to various feeding and rearing regimes [7, 8], and this complicates our understanding of the possible ways that humans managed pigs. Thus, it is unclear where, when and how pig husbandry practices developed and intensified during this complex process that eventually led domestic pigs to becoming the main animal food in human diets.

Although pig domestication was initiated c. 8500 BP in China [4, 9, 10], it is during the Yangshao period (7000–5000 BP) that domestic pigs became more prominent, as evidenced by a dramatic increase in the relative abundance of pig remains in faunal assemblages. This development is particularly noticeable in the middle Yellow River region, due to its fertile alluvial plains together with plentiful summer rainfall that favours success of pig husbandry in combination with cereal agriculture. The minimum number of individuals (MNI) and calculated meat weight (MW) of pigs reach a peak during this period, usually accounting for over 70% of faunal assemblages in the sites on the Central Plain and Wei River Plain [6]. The high frequency of pigs, combined with a decrease in body size, and high proportion of juveniles [10–13] suggest that substantial shifts occurred in pig husbandry practices during this period.

Intense pig husbandry practices would likely include control over movement, diet and reproduction [7], and here we are particularly interested in their dietary shifts induced by foddering in Neolithic farming societies. Provisioning of cereal fodder, likely consisting also of by-products including bran, leaves and stalks, is important for fattening pigs [14]. Unlike domesticated herbivore species, omnivorous pigs share a similarly broad dietary spectrum with humans, which means that they can eat human food waste and domestic kitchen scraps. Therefore, they are more conducive to close management within settlements or close to households for reuse of domestic waste. On the other hand, free-range pigs could forage in woodlands or pastures, but could also potentially raid crop fields. This is consistent with ethnographic observations of traditional pig husbandry in the Mediterranean region that pigs are kept away from growing crops [14] and an iron wire or ring in the pig’s snout is used to avoid crop damage [8]. Thus, during the process of pig domestication and husbandry, humans had to incorporate domestic pigs into their anthropogenic farming niche, finding a way in which pigs were kept away from the growing crops but meanwhile supplied with sufficient food.

In turn, pig feeding strategies closely correlate with their mode of management. Pig foddering requires confinement under close supervision, typically penned or at least tethered near the homestead. The way in which pigs were penned at a household or farmstead level can be seen in the miniature pottery sculptures of pigpens, sometimes also connected to a toilet, as burial objects found in the tombs dated to the Han dynasty (202 BC–220 AD) [15–17]. Historical records of agriculture–*Qiminyaoshu–*suggest that a seasonal mixed mode of pig husbandry was practiced in the middle and lower Yellow River region during the mid-6^th^ century AD, whereby pigs grazed in the spring and summer when plants are growing, during the autumn pigs grazed but were not foddered, and cereal bran was collected for fodder during the harsh winter and early spring [18]. The records also state that pigs naturally like aquatic weeds, that could be dragged close to the shore for fattening pigs. Thus, free-ranging and penning practices could be combined in such a pig-raising regime, based on the level of agricultural production and seasonal variation of food accessibility. The form of Neolithic pig husbandry, however, remains less clear. Given that pig husbandry practices provide a possible indication of crop surplus production, the more we know about Neolithic pig husbandry practices, the better we can understand how pigs were initially integrated into the wider agricultural system.

To explore pig feeding practices in Neolithic northern China, we determined the stable carbon (^13^C/^12^C) and nitrogen (^15^N/^14^N) isotope values of *Sus* bone collagen, to provide a proxy for pig diets under human control. Carbon isotope values (expressed as δ^13^C) of terrestrial plants are principally determined by their photosynthetic pathway [19, 20]. Most plants follow one of two dominant pathways of photosynthesis–C_3_ and C_4_ pathways–which result in distinct, non-overlapping δ^13^C values in C_3_ (−37 to −22 ‰) and C_4_ plants (−16 to −9 ‰) globally (i.e. these values incorporate the full ranges) [19, 21]. The natural ecosystem where free-ranging pigs foraged in Neolithic northern China was dominated by C_3_ plants [22–24], including trees, mostly shrubs, herbs, and root plants, whereas the cereal crops primarily cultivated in the middle Yellow River region during the Neolithic were C_4_ millets–broomcorn (*Panicum miliaceum*) and foxtail millet (*Setaria italica*) [25–31]. Thus, the isotopic composition of free-range pigs should reflect C_3_ plants, while if domestic pigs were foddered with isotopically distinct millet foods, we should be able to detect the extent of such practices.

Nitrogen isotope (δ^15^N) values of plants are determined by those of their nitrogen source, uptake mechanisms and assimilation pathways [32]. Plants that assimilate inorganic nitrogen from the soil tend to have higher δ^15^N values than those that access nitrogen through mutualistic mycorrhizae (e.g. legumes) [33]. Such isotopic differences at the base of the foodweb influence subsequent trophic levels, since there is a stepwise enrichment in ^15^N of 3 to 6 ‰ between source (diet) and consumer [34–36]. According to O’Connell et al. [35] the δ^15^N trophic step for humans falls at the higher end of these estimations (c. 5–6 ‰) and this is likely to be the case too for pigs as feeding studies suggest that pigs show a similar trophic enrichment pattern to humans [37, 38]. Anthropogenic activities impact domestic pig δ^15^N values directly, through provisioning with human food scraps, some of which are animal products [39–41], or indirectly, through the enrichment of soils surrounding settlements and in agricultural plots with organic matter, such as feces [42]. Thus, pigs under human management may be expected to exhibit higher δ^15^N values than pigs foraging in the absence of human interference.

Previous stable isotope studies have suggested that millet foddering of pigs was practiced on the Wei River Plain from the Early-Yangshao period (7000–6000 BP) [43, 44]. However, the sample sizes and number of sites explored are too limited to provide a full picture of pig management or its development through this period. In order to further understand the pig husbandry regime in the wider middle Yellow River region, here we focus on the site of Xipo, since it is a type site and regional centre in the heartland of the Yangshao Culture. Xipo underwent eight excavation seasons, from 2000 to 2013 [45–51], yielding abundant finds of a variety of archaeological features, cultural materials, faunal and floral assemblages, and an accumulation of diverse research results. Although a handful of isotopic values for Xipo pig collagen have been published previously (δ^13^C, n = 6; δ^15^N, n = 2) [52, 53], they are too few to be able to discuss local pig management and its development in relation to farming practices in detail. Further excavations have since been carried out at Xipo, thus providing a much larger sample of faunal remains from more extensive contexts and allowing us to explore pig feeding practices and understand the pattern and intensity of pig husbandry practices at this key site.

In this study, we first explore the diets of domestic pigs at Xipo based on the stable isotopic composition of bone collagen, and we then compare these results against those published for humans from the Xipo cemetery. Next we compare the Xipo results with those of pigs from other Yangshao sites in the middle Yellow River region, aiming to:

- Understand the pig feeding practices and management modes at Xipo;
- Disentangle the relationships between pig husbandry, human diets and local farming;
- Gain greater insights into pig husbandry regimes in the middle Yellow River region during the Yangshao period.

## 2. Archaeological context

### 2.1 Study site

The Yangshao Culture (7000–5000 BP) is characterised by distinct painted ceramics, the appearance of large, densely populated settlements with sophisticated layouts, and a mixed mode of human subsistence [54]. These cultural attributes were shared across farming societies in the middle Yellow River region. Due to its significant influence on both contemporaneous and succeeding Neolithic cultures, this period is known as the Yangshao Cultural Period. Its prosperity was based on an economy consisting of millet cultivation and pig farming, supplemented with hunting and gathering. More than 2500 sites were found in the Shaanxi and Henan provinces for the whole Yangshao Cultural Period [55], suggestive of substantial human population growth fostered by a successful economy. During its middle phase (6000–5500 BP), the Yangshao Culture developed rapidly and expanded beyond its original geographic range, strongly influencing peripheral regions in terms of both material culture and subsistence economy [54, 56], and reaching its peak of prosperity.

**Fig 1:**
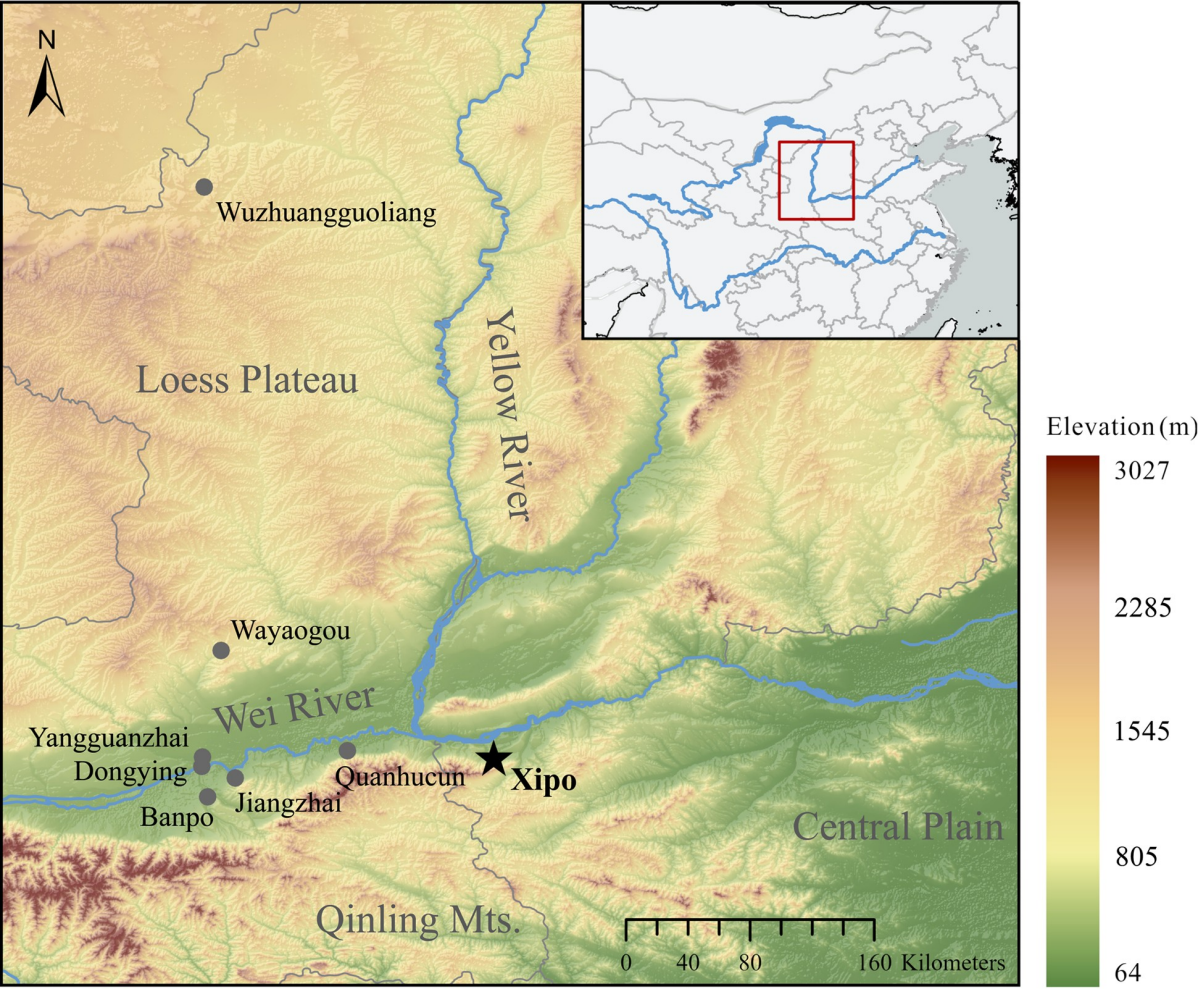
Map of Xipo and the other Yangshao Culture sites in the middle Yellow River region with published pig stable isotope data or mentioned in this study. Map prepared by Qi Meng in ArchGIS Pro 2.5.0 using Global Multi-resolution Terrain Elevation Data 2010 (GMTED2010) from USGS EROS [58].

**Fig 2:**
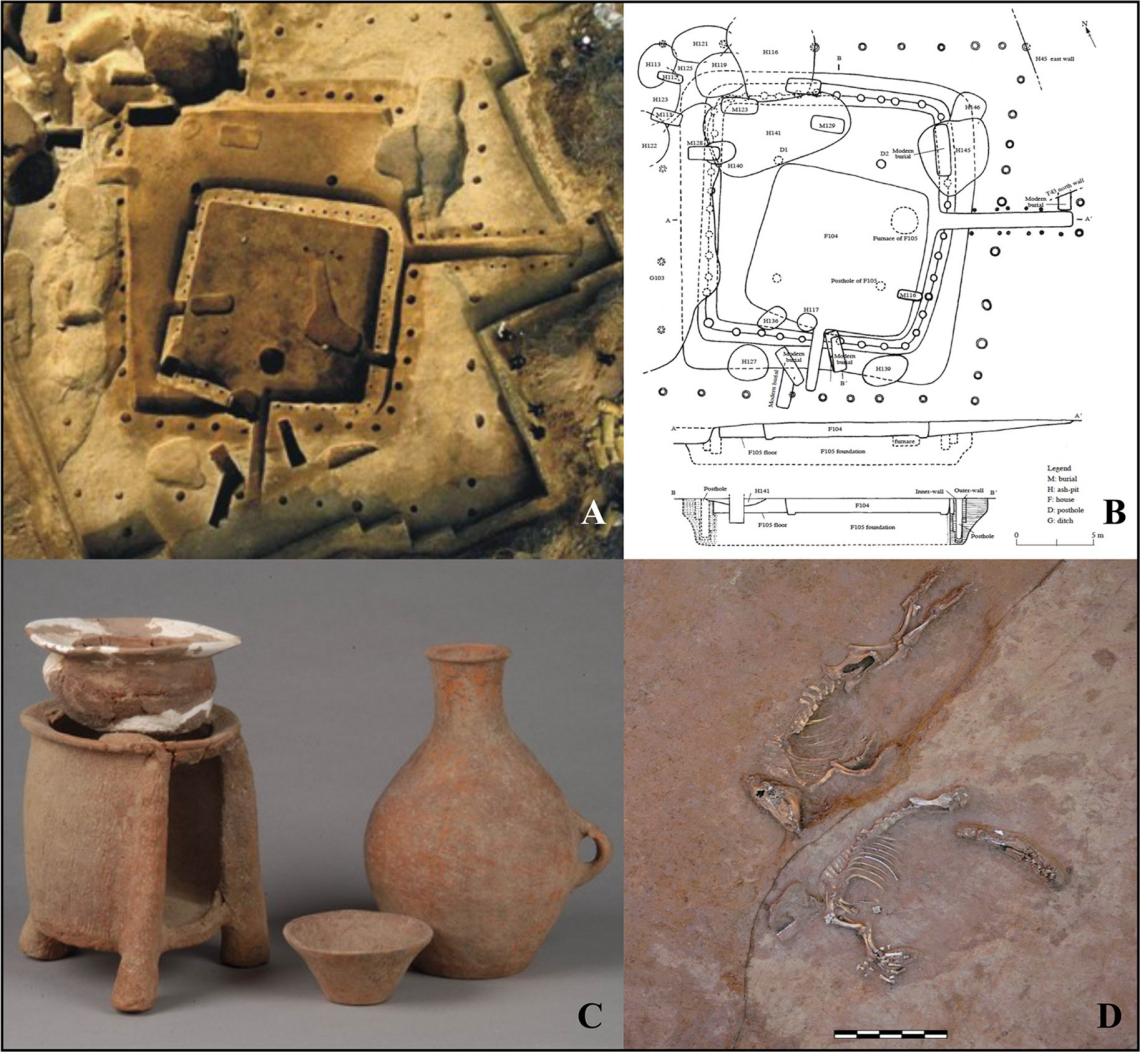
Communal buildings, ceramics and pig individuals discovered from the Xipo site. (A) Photograph of the houses F105 and F104. (B) Plan of the houses F105 and F104 [45]. (C) A set of ceramics buried in M13 in the Xipo cemetery. (D) Two pig individuals recovered at the bottom of the moat [50]. (Photograph courtesy of Xinwei Li).

Among the sites dating to the Middle-Yangshao period, Xipo exhibits a typical material culture in the heartland of the Yangshao Culture’s distribution. It is located on a fertile terrace between the middle Yellow River and the eastern edge of the Qinling Mountains, on a passageway bridging the Wei River Plain and the Central Plain (Fig 1). It was occupied from 5800–5000 BP and covers an extensive area of c. 40 hectares [57]. Due to its large size, it is considered to have been a regional centre of the Middle-Yangshao Culture Miaodigou Type [13]. It is distinguished from other smaller settlements by the presence of several large-scale communal buildings over 200 m^2^ in size in the central area (F105 over 500 m^2^ including surrounding corridor) (Fig 2A and 2B), constructed surrounding moats, a cemetery (5300–5000 BP) with arranged burials, and uneven distribution of exquisite burial objects, jointly suggesting the emergence of increasing social inequality and complexity [47, 57]. A distinctive feature of the site is that the large houses discovered in the central area are believed to have accommodated public activities, such as ritual, funeral and feasting events [57]. However, no evidence has been found for the accumulation of faunal refuse dumps directly associated with large feasting events held in the large houses. Thus, the feasting events, if they occurred, were likely not on a large scale, and activities may have included other types of rituals or public gatherings.

According to the recovered plant remains, stable isotope values of human bones, and starch grain analyses of dental calculus [47, 59], foxtail and broomcorn millets were the primary cereal crops consumed by humans. Limited numbers of rice (*Oryza sativa*) and soybean (*Glycine max*) remains were also recovered. Many of the ceramics recovered from the burials in the Xipo cemetery are clearly functional and assumed to be associated with the cooking of millet (Fig 2C). Pigs dominate the faunal assemblage, accounting for 84% of the number of identified specimens (NISP) with a high proportion (90%) of juveniles, and are argued to have been the main animal protein source in human diets [13]. The high concentration of pig remains (MNI = 23–53) in some large refuse pits near medium-sized buildings (75–106 m^2^) suggests that pigs were likely consumed during feasting events held in or near these buildings [13]. Articulated skeletons of two pigs were found at the bottom of the moat (Fig 2D) and several articulated skeletons of neonatal pig individuals and piglets were also recovered at the site. There were no pig remains found buried with humans in the Xipo cemetery. The very large Xipo pig assemblage is, we argue, a reflection of a strong concentration on pig husbandry that represents the intensification of this practice, and hence is worth exploring in more detail.

## 3. Materials and methods

### 3.1 Archaeological samples from Xipo

In total, bone specimens of 70 *Sus* and 17 other faunal species were selected for stable isotope analysis (S1 Table). The *Sus* samples were taken from adult mandibles, where available, and collected from a variety of archaeological contexts, including refuse pits, houses, moats, kilns and cultural layers, to test for potential spatial variation in the raising of pigs. Other faunal species analysed include dog (*Canis*), cattle (*Bos*), sheep/goat (*Ovis/Capra*), deer (*Cervus*), bear (*Ursus*), and bird (*Avis*), which were selected to compare the diets of domestic animals and to establish an isotopic baseline of the local ‘wild’ ecosystem. Cattle and dog specimens came from contexts dated to the later Western Zhou period (1046–771 BC). Details of the contexts and phases of animal bone samples are given in S1 Table.

Permission to perform the analysis of the faunal materials from Xipo was issued by the National Cultural Heritage Administration, China (permits [2011] No.136; [2013] No.64). The specimens are stored in a repository of Henan Provincial Institute of Cultural Heritage and Archaeology in Zhengzhou, Henan, China, which are not publically accessible. For research purposes, any individuals or groups can seek access to the materials from this institution, with support from the excavation co-director (Dr. Xinwei Li). This described study complied with all relevant regulations.

### 3.2 Preparation of bone collagen and stable isotope analysis

Bone collagen was extracted following a standardised protocol [60]. Approximately 500 mg of bone chunks were demineralised with 0.5 M HCL at 4°C until they were completely decalcified. After rinsing with deionised water, samples were treated with 0.1 M NaOH for 30 mins to remove potential humic acid contaminants. After rinsing thoroughly to neutrality, samples were gelatinised in pH3 solution at 70°C for 48 hours. Finally, the resulting solutions were frozen and freeze-dried for 48 hours to produce dry bone collagen.

For stable isotope analysis, approximately 1 mg of bone collagen for each sample was weighed into tin capsules to determine the ^13^C/^12^C and ^15^N/^14^N ratios using a Sercon 20/22 continuous flow mass spectrometer coupled to a Callisto elemental analyser. Stable carbon and nitrogen isotope ratios are expressed using the delta (δ) notation as:

δ13Corδ15N(000)=(Rsample−RstandardRstandard)×1000

where R = ^13^C/^12^C or ^15^N/^14^N. The δ^13^C and δ^15^N values are reported relative to the international Vienna PeeDee Belemnite (VPDB) and Ambient Inhalable Reservoir (AIR) standards, respectively. An internal standard, Alanine, and two further internal bone collagen reference materials, COW and SEAL, all previously calibrated to international reference materials were included in each run to correct for drift, to monitor measurement uncertainty and to calibrate the raw data. Precision of measurement was calculated as ±0.08 ‰ for δ^13^C and ±0.12 ‰ for δ^15^N based on multiple measurements of the collagen standards. Accuracy was determined to be ±0.10 for δ^13^C and ±0.16 for δ^15^N based on the difference between the observed and known δ values of the standard Alanine and its long-term standard deviation. The total analytical uncertainty was estimated to be ±0.13 for δ^13^C and ±0.2 for δ^15^N using the equations given by Szpak et al. [61]. Additional details are provided in S1 Table.

The state of collagen preservation was assessed by collagen yield, weight percentage of C and N and the atomic C:N ratio [62]. The C:N ratio is considered to be a robust quality control indicator for collagen and the typically acceptable range for archaeological bone collagen is from 3.1 to 3.5 [63, 64]. A recent study comparing C:N ratios against fluorescence methods showed a narrower range of 3.1 to 3.3 for demonstrably well preserved collagen [65], which is also close to the theoretical yield of 3.2 for intact collagen [66].

## 4. Results

### 4.1 Faunal stable isotope values from Xipo

**Fig 3:**
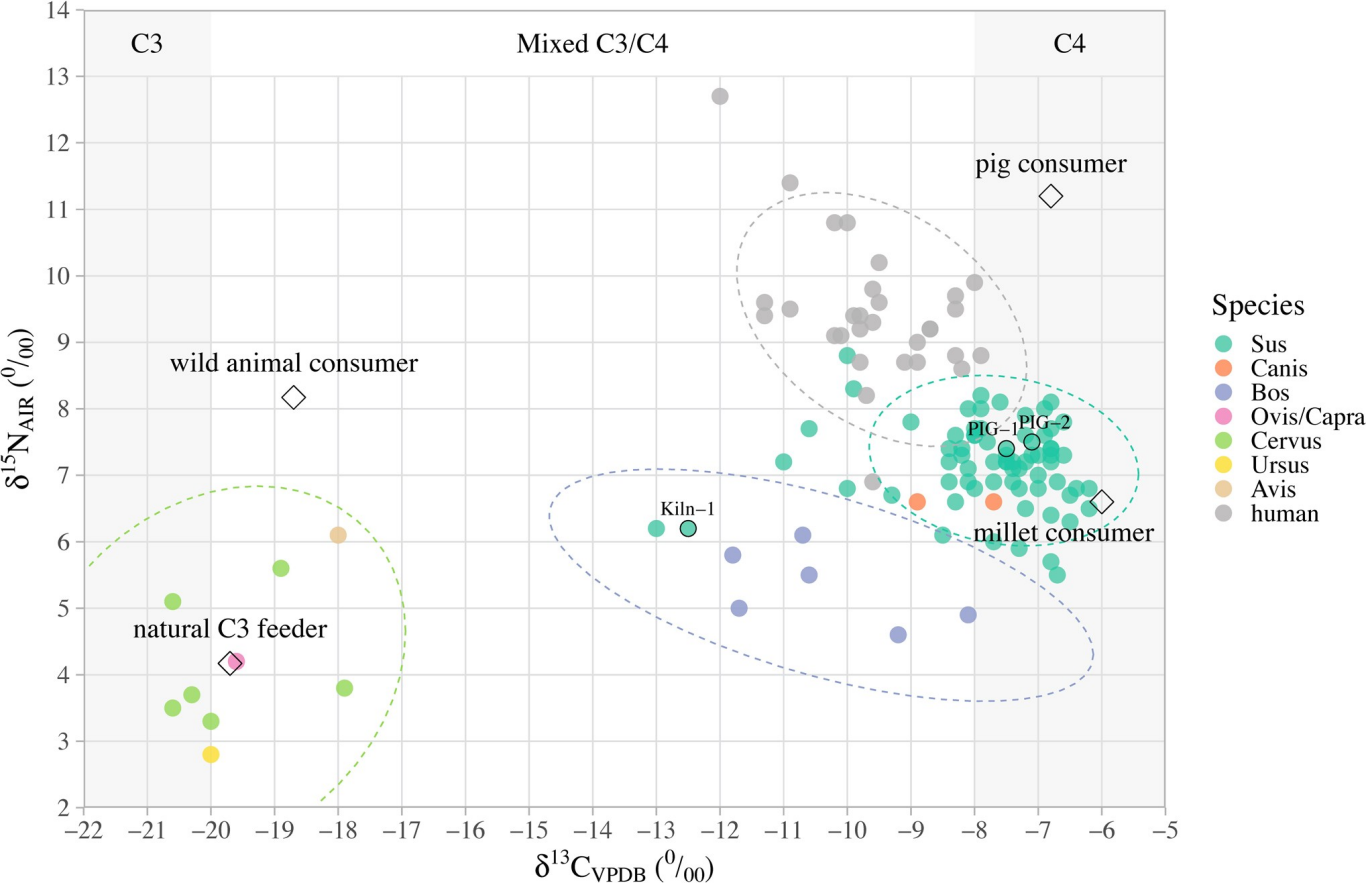
Bi-variate scatter plot of the δ^13^C and δ^15^N values of animal and human bone collagen from Xipo. The predictive ranges for C_3_, mixed and C_4_ dietary groups are determined by the mean values of C_3_ plants [19, 21] and C_4_ millets [67, 68], plus ^13^C enrichment of 5 ‰ between diet protein and collagen [69, 70]. Human isotopic data are from the Institute of Archaeology CASS and Henan Provincial Institute of Cultural Heritage and Archaeology [47]. 95% prediction confidence ellipses are drawn for *Sus*, *Bos*, *Cervus* and humans; other species have too few data points. ‘PIG-1’ and ‘PIG-2’ were recovered from the bottom of the moat. ‘Kiln-1’ was recovered associated with the ceramic kiln. The modelled values for different consumer groups are symbolised by open diamonds and calculated as follows. The pure ‘millet consumer’ is based on published millet mean δ^13^C values + a stepwise diet-consumer collagen shift of +5 ‰, and mean δ^15^N value with an estimated trophic step of + 4 ‰. For ‘pig consumer’ we used the Xipo pig mean δ^13^C + 1 ‰ as the trophic step for δ^13^C, and mean δ^15^N + 4 ‰ as the trophic shift. For ‘natural C_3_ feeder’, we used the mean δ^13^C and δ^15^N for wild herbivores and for any wild animal consumers we used the latter + 1 ‰ and + 4 ‰ for δ^13^C and δ^15^N respectively.

The δ^13^C and δ^15^N values for all faunal samples from Xipo are shown as a bivariate plot in Fig 3 and summarised for each species in Table 1. The data and quality control indicators are provided in S1 Table; all Xipo collagen samples fell in the C:N range of 3.2 to 3.4.

**Table 1:** Descriptive statistics of faunal bone collagen δ^13^C and δ^15^N data at Xipo.

	Species	δ^13^C			δ^15^N			n
		Mean	SD	Range	Mean	SD	Range	
*Domestic*	*Sus*	−7.8	1.3	−13.0 to −6.2	7.2	0.6	5.5 to 8.8	70
	*Canis*	−8.3	0.8	−8.9 to −7.7	6.6	0	6.6 to 6.6	2
	*Bos*	−10.4	1.4	−11.8 to −8.1	5.3	0.6	4.6 to 6.1	6
*Wild*	*Cervus*	−19.7	1.1	−20.6 to −17.9	4.2	0.9	3.3 to 5.6	6
	*Ovis/Capra*	−19.6	*NA*	*NA*	4.2	*NA*	*NA*	1
	*Ursus*	−20.0	*NA*	*NA*	2.8	*NA*	*NA*	1
	*Avis*	−18.0	*NA*	*NA*	6.1	*NA*	*NA*	1

*Cervus* specimens show a mean δ^13^C value of −19.7±1.1 ‰ (n = 6), similar to δ^13^C values for other wild fauna, including *Ovis/Capra* (−19.6 ‰), *Ursus* (−20.0 ‰) and *Avis* (−18.0 ‰) and indicating that wild herbivores foraged in similar open C_3_ environments in the vicinity. *Cervus* δ^15^N values range from 3.3 to 5.6 ‰ (mean 4.2±0.9 ‰) and that of the single ovicaprid is 4.2 ‰. These values likely reflect the baseline range of δ^15^N values for herbivores feeding in an undisturbed wild environment. Pigs have the highest δ^13^C and δ^15^N values of all of the fauna sampled (Table 1). Compared with the wild herbivores, the δ^13^C and δ^15^N values of pigs (n = 70) range widely, from −13.0 to −6.2 ‰ (−7.8±1.3 ‰) and from 5.5 to 8.8 ‰ (7.2±0.6 ‰), respectively, indicating that most pigs had an omnivorous diet that included significant amounts of C_4_ resources. A few pigs exhibited δ^13^C values below −8 ‰, suggesting that they also consumed some C_3_ foods. We found significant differences between the wild herbivores and domesticated pigs for both δ^13^C (T-test: t = 23.3, df = 75, p-value < 0.001) and δ^15^N values (T-test: t = 11.5, df = 75, p-value < 0.001). Two other domestic animals, dogs (n = 2) and cattle (n = 6), are from the later Western Zhou period. The dogs with δ^13^C values of −7.7 ‰ and −8.9 ‰, and δ^15^N values of both 6.6 ‰ are very similar to the range of pigs, while the cattle show δ^13^C (−10.4±1.4 ‰) and δ^15^N values (5.3±0.6 ‰) lower than those of the pigs.

## 5. Discussion

### 5.1 Pig diets at Xipo

The majority of pigs have δ^13^C values clustered between −8.5 and −6 ‰ (Fig 3), indicating a dominance of C_4_ carbon in their diets. Since the main source of C_4_ plants was cultivated millets, these pigs were predominately fed millet foods or millet derived by-products, thus we can infer that they were closely managed by humans. The mean δ^13^C values of charred millet grains recovered from the middle Yellow River region during this period range from c. −10 to −11 ‰ [67, 68]. The modelled mean δ^13^C and δ^15^N values of the pure millet consumer (labelled ‘millet consumer’ in Fig 3) are calculated based on the published millet mean δ^13^C values + a stepwise diet-consumer collagen shift of +5 ‰ [69, 70], and mean δ^15^N value + a stepwise diet-consumer collagen shift of + 4 ‰ (a mid-point of the values determined by [34–36]). The pig data are close to the modelled isotopic values for a mostly millet consumer (Fig 3), with a minor dietary contribution of C_3_ resources. These results are consistent with the conclusions of Pechenkina et al. [52] and Zhang et al. [53]: millet fodder was likely fed to pigs. Notably, millet leaves are ^13^C-depleted by approximately 1 ‰ compared to grains [67, 71]. It is therefore plausible that the δ^13^C values of most Xipo pigs reflect consumption of millet by-products, but it isn’t possible to infer grain vs leaf/stalk dietary contributions within a possible mixture of C_4_ and C_3_ resources.

No pigs were found to have diets dominated by C_3_ or even diets comprising >50% C_3_ resources (Fig 3). This result strongly suggests that no free ranging wild boar were present in the sample set. Since this is a substantial number of samples, we infer that there is very little evidence for the presence of wild boar at the site. A small group with δ^13^C values below or near −9 ‰ evidently consumed a mix of C_4_ and C_3_ foods in varying proportions; they may have been partially free-ranging, or confined pigs fed some C_3_ foods as well as millets. Inter-seasonal and annual variation in abundance of millet foods and diverse inputs from human food waste likely account for the observed variation in pig δ^13^C values.

Pigs and dogs, both of which show the highest proportion of C_4_ consumption of all animals, also show the highest δ^15^N values, especially when compared with wild fauna. There are two possible explanations. One is a higher contribution of animal protein to their diet, likely through provision of household waste. Alternatively, cultivated millets may have had higher δ^15^N values than wild plants, due to differences in the soils in which the crops were grown, and/or deliberate addition of organic matter from settlements. The second possibility is consistent with the observation that *all* domestic animals have higher δ^15^N values than wild fauna, suggesting that the areas in which domestic fauna were herded were affected by anthropogenic inputs. For instance, cereal stubble after harvest could provide good grazing for pigs, while their feces could contribute to enrichment of the soils.

In terms of the spatial distribution of pigs, two were recovered from the bottom of the moat (Fig 2D), and have been considered as possible ritual offerings [50]. Their δ^13^C and δ^15^N values (δ^13^C = −7.5, −7.1‰; δ^15^N = 7.4, 7.5‰), however, fall squarely into the middle of the range for all pigs (Fig 3). No obvious spatial differences in pig diet is detected, but some inter-individual variation is observed. One pig recovered in association with the kiln shows relatively low δ^13^C and δ^15^N values of −12.5 ‰ and 6.2 ‰ (Fig 3), suggestive of a mixed C_3_/C_4_ diet. Considering that ceramic production required a huge amount of wood fuel, possible explanations include that this pig was allowed to partially free-range in the woodland under extensive management, or that this pig was a wild one encountered during the collection of wood from the woodland in the vicinity of the kiln and then foddered for a period. The other pig with a similarly low δ^13^C value, however, was not from a particular context. Therefore, there is no clear relationship between low δ^13^C values and contexts associated with woodland use.

Hence, at Xipo, almost all the pigs analysed were almost exclusively fed millet by-products, and thus they were under close human management. The controlled feeding regime suggested by the isotope data must have involved substantial labour inputs in pig husbandry alongside an expanding human population. Meanwhile, this Middle-Yangshao period coincided with high mean summer precipitation that peaked in 6100−5500 BP [68]. This was also a time of rain-fed millet agriculture expansion in northern China [27], in which we propose that high yields and surplus of millet foods and by-products had become available to feed larger numbers of domestic pigs. Pig feeding practices thus provide a link to surplus production and the availability of crop fodder for animal feed, and also confirm that a mutualism between millet agriculture and animal husbandry was likely established [52]. The data strongly suggest an intensification of pig husbandry co-incident with increased prosperity of the Middle-Yangshao period based on millets as a staple crop [54].

In order to further understand other aspects of pig husbandry at Xipo, we will extend the isotope research to bioapatite, since collagen emphasises the protein component of diets, while analyses on bioapatite should provide a more nuanced indication of the carbohydrate components, if they were distinct. Especially high-resolution methods, i.e. sequential sampling of tooth enamel, should be able to track seasonal dietary variations and detect some special dietary episodes.

### 5.2 Comparison of pig and human diets at Xipo

To better understand the relationship between pig and human diets, we compared the bone collagen isotopic data for the pigs against published data for humans from the Xipo cemetery [53]. The humans have mean δ^13^C and δ^15^N values of −9.6±1.0 ‰ and 9.4±1.0 ‰ (n = 30), indicative of omnivorous diets with a significant contribution of C_4_ resources, i.e. millets. Referring to the modelled values of the pure millet consumer, the distribution of δ^13^C and δ^15^N data suggests that most pigs ate more millet foods than humans (Fig 3). This may suggest that a surplus of millet by-products was used for animal feed. At the same time there is no evidence to suggest that millets thus held a lower status for humans, as humans were evidently also consuming millets, and humans and pigs were potentially using different parts of the same plants.

On average, compared to pigs, humans are enriched in ^15^N by +2.3 ‰ but depleted in ^13^C by 1.8 ‰. Trophic level studies of simple prey-predator systems normally show an enrichment of 1 ‰ in ^13^C for every 3 ‰ enrichment in ^15^N [72]. For simplification, we use + 1 ‰ as the trophic step for δ^13^C. The modelled δ^13^C and δ^15^N values of the pure ‘pig consumer’ are calculated as −6.8 ‰ and 11.7 ‰. These modelled δ^13^C and δ^15^N values are both higher than the actual human mean values (Fig 3). These observed differences are consistent with some human consumption of pig meat, but the relatively low δ^15^N values of humans compared to the modelled δ^15^N value of a pure ‘pig consumer’ shows that pigs made a modest contribution to the human diet. Together with the more negative human δ^13^C values, it strongly suggests that in addition to millets and pigs, a different source of food was also consumed–one that has a significantly lower δ^13^C value. Likely candidates would be C_3_ plants (e.g. rice, nuts, fruits, tubers, lotus roots, etc.) and/or animals that consumed C_3_ plants, like wild fauna (e.g. *Cervus*, *Ovis/Capra*, *Rhizomys*, etc.).

**Fig 4:**
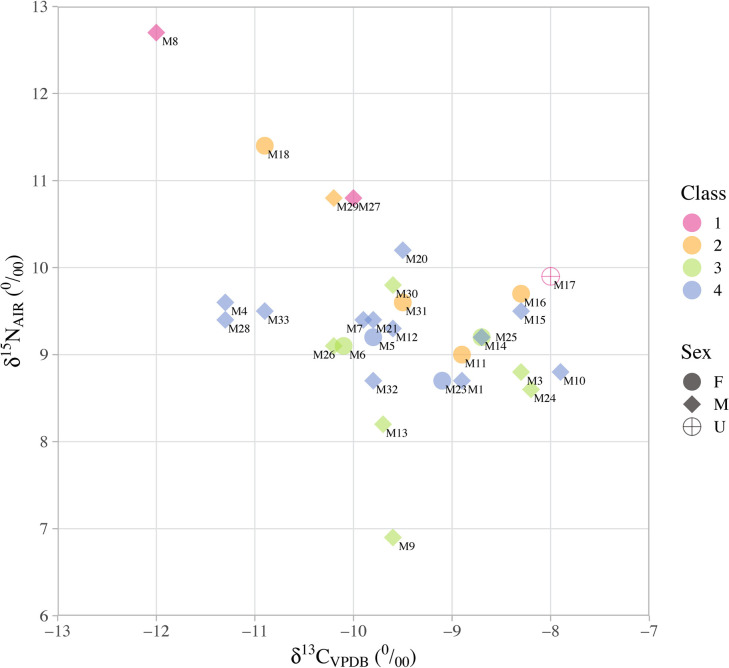
Bi-variate scatter plot of the δ^13^C and δ^15^N values of human bone collagen from the Xipo cemetery against their sex and burial class (data from Institute of Archaeology, CASS and Henan Provincial Institute of Cultural Heritage and Archaeology [47]). Classes 1–4 are described above and detailed in S2 Table. Sex of the burial owners is presented as female (F), male (M) and unidentified (U).

**Fig 5:**
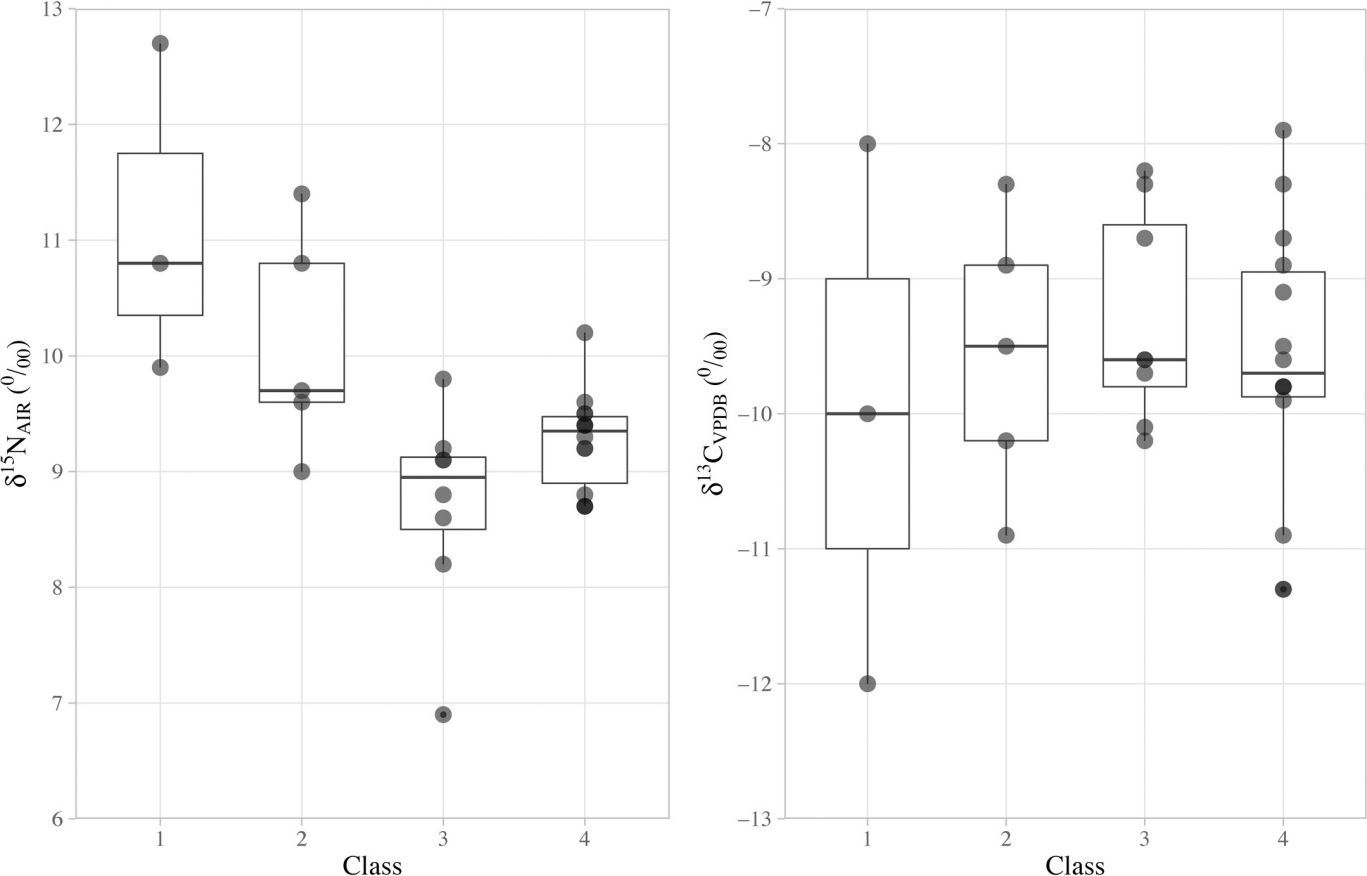
Box plot of the δ^15^N and δ^13^C values of human bone collagen from the Xipo cemetery against their burial class, respectively (data from Institute of Archaeology, CASS and Henan Provincial Institute of Cultural Heritage and Archaeology [47]). Classes 1–4 are described above and detailed in S2 Table.

We further explored the relationship between human diets and social status by a re-analysis of the published collagen isotopic results for Xipo humans from the cemetery, against a hierarchy of four burial groups (Figs 4 and 5). The burial classes are grouped according to the burial dimensions, contained burial objects and their type values, which in part at least reflect the wealth and social status of their owners [47]. Class 1 is ranked as the ‘highest status’, and Class 4 as the ‘lowest status’ in the hierarchical sequence. The detailed information of burial classes is provided in S2 Table.

In order to explore the relationship between human diets and social status, we use ordered logistic regression (performed in RStudio 1.1.463, package MASS, function polr, see S3 Table) to test whether human δ^15^N and δ^13^C values are correlated with their burial class. The results show that human δ^15^N values are correlated with their assigned burial class with statistical significance (p = 0.01), but no clear differentiation in δ^13^C values between ‘high’ and ‘low’ status individuals (p = 0.20) is apparent (Fig 5). In terms of the δ^15^N data, the dietary ‘distances’ between these burial classes are not equal. For instance, the differentiation between Class 1 and Class 2 (p = 0.005) is larger than that between Class 2 and Class 3 (p = 0.01) and also between Class 3 and Class 4 (p = 0.02). Specifically, the high-status people with large graves and more valuable grave objects tend to have higher δ^15^N values than those with small graves and few grave goods. The most parsimonious explanation is that these high-status individuals consumed more animal protein and the animal source was most likely pig meat since wild animals have significantly lower δ^15^N values. Together these data suggest that high-status people had preferential access to protein sources enriched in ^15^N compared to lower-status individuals, but there is no significant difference in carbon isotope values. Therefore, this isotopic evidence suggests that millets were eaten by both high and low status individuals, rather than being reserved for individuals of a certain social status.

In particular, one male from grave M8, assigned to burial class 1, shows the highest δ^15^N (12.7 ‰) and lowest δ^13^C value (−12.0 ‰) of all individuals published. It seems that in addition to millets and pigs, some C_3_ food sources led to his bone collagen exhibiting a more negative δ^13^C value and higher δ^15^N value. Eating hunted wild herbivores does not appear to be a plausible explanation, however, since those analysed in this study show significantly lower δ^15^N values than domestic pigs (Fig 3). Thus, some C_3_ foods with higher δ^15^N values in the diet of this high-status male are likely missing from this stable isotope analysis. This might be a source such as freshwater fish or possibly ^15^N-enriched vegetables in manured gardens. These results hint at a relationship between grave classes, isotopic composition, diet and possibly lifestyle, which requires further investigation.

### 5.3 Pig diets during the Yangshao period in the middle Yellow River region

**Fig 6:**
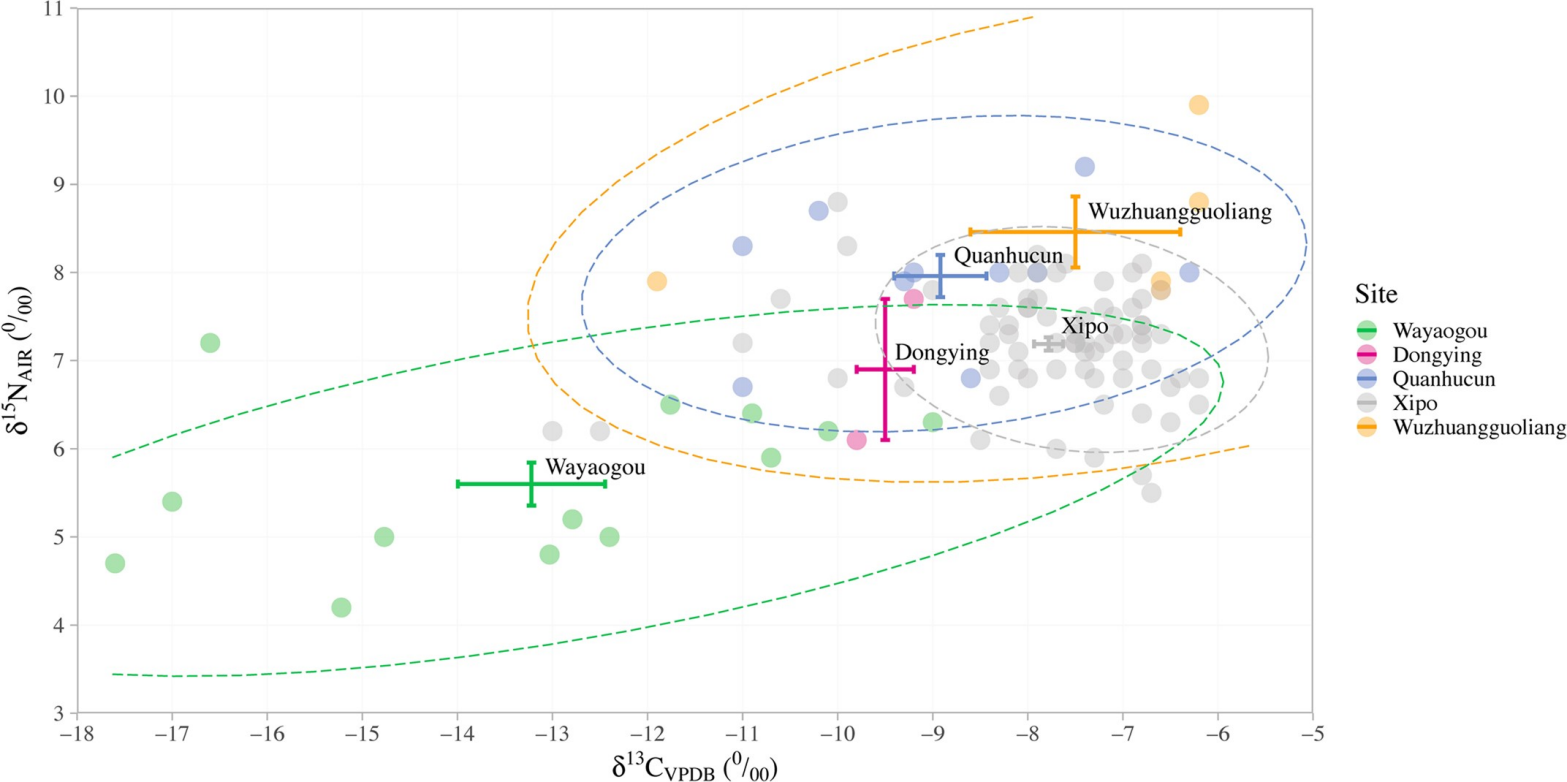
Bi-variate scatter and error bar plot of the δ^13^C and δ^15^N values of pig bone collagen from the Yangshao sites in the middle Yellow River region. The order of the five sites listed in the legend coincides with their chronological sequence from the oldest to youngest. Error bars represent the mean δ^13^C and δ^15^N values ± 1SE for each site, and 95% prediction confidence ellipses are calculated for each site except for Dongying, which has too few data points.

In order to understand diachronic shifts in pig diets and hence husbandry during the entire Yangshao period in the middle Yellow River region, pig data from Xipo were compared with published data from other Yangshao sites, as shown in Fig 6.

Notably, both the δ^13^C and δ^15^N values of pigs increased through time with statistical significance (Ordered Logistic Regression δ^15^N: p<0.001; δ^13^C: p = 0.04, see S3 Table), indicative of a diachronic trend of increasing millet food consumption, and increasing δ^15^N values suggestive of higher trophic diets. The pigs from the Wayaogou site (6500–6000 BP), having mean δ^13^C and δ^15^N values of −13.2±2.8 ‰ and 5.6±0.9 ‰ (n = 13) [43, 44], and suggesting mixed C_3_/C_4_ diets. This indicates that from the Early-Yangshao period pigs were partly fed with millet, and in varying proportions. Dongying (5900–5600 BP) has too few datapoints, but the available pig δ^13^C values from this site are also consistent with a significant contribution of millet foods in their diets [43]. The Quanhucun site of the Middle-Yangshao Culture is contemporaneous with Xipo. The δ^13^C and δ^15^N values of Quanhucun pigs largely overlap with those of Xipo pigs, but show slightly more negative δ^13^C values and higher δ^15^N values (−8.9±1.5 ‰; 8.0±0.8 ‰; n = 10) [73]. In the Late-Yangshao period (5500–5000 BP), pigs from the Wuzhuangguoliang site on the Loess Plateau have the highest δ^13^C and δ^15^N values of −7.5±2.5 ‰ and 8.5±0.9 ‰ (n = 5) [74]. Their diets were strongly dominated by millets.

Among these Yangshao sites in the middle Yellow River region, Xipo pigs display the most tightly clustered values with the least variation. This suggests uniform diets with a relatively narrow range of food sources, and which we infer were strongly controlled by humans. Compared with the other Yangshao sites, Xipo pig δ^13^C and δ^15^N values hint at the presence of some C_3_ foods with elevated δ^15^N values in their diets (Fig 6), just like in those of their owners. These C_3_ foods were possibly derived from human food scraps. Overall, however, the relatively high δ^13^C values of pigs from the three Middle-Yangshao sites show that foddering pigs with millet foods intensified in this period, which coincided with the great success of millet agriculture in the middle Yellow River region and also with the climax of the Yangshao Culture.

### 5.4 Pig husbandry at Xipo

The stable isotopic results suggest that Xipo pigs had a predominantly C_4_ diet as a result of intensive millet feeding, and thus it is likely that they had been kept in confinement or under close management for most of their lifetime. The main form of pig control (in addition to foddering) would be penning. Some small walled features at Xipo have been interpreted as possible pigpens [52], but no definitive evidence has yet emerged. Some evidence of penning was also found dating to the Early-Yangshao period in the middle Yellow River region, such as at the Jiangzhai and Banpo sites on the Wei River plain (Fig 1) [75, 76]. Here, architectural evidence of earthen structures, interpreted as possible pens, was uncovered in the central area of these settlements, perhaps suggesting that domestic pigs at this time were reared in a communal way rather than at a household level. Intensive pig foddering would likely have required the collective gathering and storage of large quantities of agricultural by-products for pig feed. Consequently, some members of society may have been heavily engaged in supporting pig husbandry.

Pigs were also used in diverse ritual activities. The burial of pig remains, particularly mandibles, along with humans in graves, is a frequently observed phenomenon initiated from the Pre-Yangshao period c. 8500 BP onwards [4, 9, 77]. By the Middle-Yangshao period, however, pigs were no longer placed in burials in the middle Yellow River region [6], as seen in the type sites of this period, such as Xipo and Yangguanzhai. This change implies a subtle shift in the conceptualisation of pigs at this time, such as ownership. By this stage pigs may have been seen more as a resource or commodity rather than as indicative of wealth or status. However, pigs were still used in some ritual contexts. At Xipo, the burial of two pigs at the bottom of the moat (Fig 2D) might suggest a relationship to the moat construction ceremony, comparable to the phenomenon of burying animals in house foundations. However, there is no evidence to suggest that these pigs believed to have been used for rituals had special diets. Given that pig remains dominate the faunal assemblages, it is surprising to note that there is little strong evidence for their use in ritual activities [78].

In spite of the strong dependence in these Yangshao sites on pig husbandry, there is a paucity of strong evidence for built features, such as pens, indicative of local pig raising. Yet compared with other Yangshao sites in the middle Yellow River region, the relatively narrow range of isotopic data for Xipo pigs indicates that a relatively homogenous pig foddering regime was employed, suggesting that the pigs were raised *in situ* rather than brought to the site from a hinterland. We suggest that pig husbandry practices at Xipo were predicated on taking advantage of crop surplus production, and were tightly integrated into the millet farming system.

## 6. Conclusion

Isotopic data from the site of Xipo show that pigs were intensively fed on millets, along with other minor sources such as household waste. This observation suggests that they were confined within or close to the Xipo settlement in close proximity to humans. The intensive foddering strategy for fattening pigs supported the growing human population, and at the same time demanded a high labour input. In spite of the evident economic importance of pig husbandry and close proximity of pigs and humans, the evidence for their use in ritual activities is, so far, surprisingly limited. Certainly, if they were used for such activities, the isotopic data lacks evidence for the presence of any specially fed pigs. Comparisons with other Yangshao sites elsewhere in the middle Yellow River region suggest a trend towards intensified pig husbandry practices, particularly millet foddering, during the Yangshao period. From the Middle-Yangshao phase, millets became the predominant pig fodder, which were likely derived from crop surpluses. Together, the importance of millet foods in pig foddering and pig consumption in human diets at Xipo indicates that pigs became an integral part of both the local farming regime and subsistence economy in the middle Yellow River region from this period.

## Supporting information

S1 Table Faunal bone collagen δ^13^C and δ^15^N values and sample contextual information. Details of instrument measurement, data calibration, uncertainty calculation, collagen yield and sample information.
(XLSX)

S2 Table Burial classes in the Xipo cemetery. (XLSX)

S3 Table Statistical analyses. Ordered Logistic Regression in 5.2 and 5.3.
(XLSX)
